# Investigating the Viability of Motor Imagery as a Physical Rehabilitation Treatment for Patients With Stroke-Induced Motor Cortical Damage

**DOI:** 10.7759/cureus.14001

**Published:** 2021-03-19

**Authors:** Asavari S Gowda, Areeba N Memon, Erjola Bidika, Marina Salib, Bhavana Rallabhandi, Hafsa Fayyaz

**Affiliations:** 1 Medicine, California Institute of Behavioral Neurosciences & Psychology, Fairfield, USA; 2 Internal Medicine, California Institute of Behavioral Neurosciences & Psychology, Fairfield, USA; 3 Neurology, California Institute of Behavioral Neurosciences & Psychology, Fairfield, USA

**Keywords:** mirror neurons, neurological connectivity, stroke, motor cortex, motor cortical, infarction, plasticity, motor imagery, motor execution, m1

## Abstract

Although around 83% of individuals survive a stroke, they usually experience a significant loss in their motor execution (ME) capabilities due to their acquired cortical infarction. The loss of significant ME capabilities due to stroke damage was previously thought to be irreversible. Active movement therapies show considerable promise but depend on motor performance, excluding many otherwise eligible patients. Motor imagery (MI), a process that involves the use of mirror neurons to imagine motor activity, has emerged as a possible avenue to re-acquire some physical abilities lost to stroke damage. This paper examines previous studies to compare the strength of brain activation and connectivity in individuals who have brain lesions and those who do not as they all attempt ME and MI tasks. This paper reviews case studies investigating the direct effect of motor imagery in conjunction with physical therapy and the limitations of motor imagery based on the location of cortical damage and other variables, such as age. The findings analyzed in this review indicate that MI would serve as a beneficial addition to physical therapy and a viable option to stimulate motor evoked potentials (MEPs) in individuals not capable of pursuing physical therapy due to severe motor impairment. Regardless of the presence of brain lesions, motor imagery has consistently had a positive impact on motor rehabilitation either in boosting treatment or stimulating neuromuscular pathways. Therefore, we have concluded that MI is a viable supplemental treatment plan for motor recovery in most patients with motor cortical atrophy.

## Introduction and background

Of the 795,000 people around the world who experience a stroke each year, about 658,000 survive [1]. However, survivors of stroke usually have significant cortical damage that results in paralysis on the side of the body contralateral to the lesioned hemisphere [2]. Thus, most stroke rehabilitation methods are directed towards helping the individual regain their physical abilities. There are several therapies used on patients to improve motor skills they lost from stroke damage. Active movement therapies show considerable promise but depend on motor performance, excluding many otherwise eligible patients [3]. Motor imagery (MI) is a rehabilitation method that has gained traction over the past several years. It’s an emerging form of treatment that involves the patient imagining the movement they intend on performing and then attempting to physically execute the movement. It has previously been used to improve athletic performance but has recently been shown to help patients reacquire lost motor abilities from cortical damage [4].

The motor cortex is an important structure for any motor movement targeted rehabilitation treatment. However, its role, and the role of a human cerebral cortex as a whole, in motor imagery is still being researched. The motor cortex is made up of four parts: primary motor cortex (M1), supplementary motor area (SMA), premotor area (PMA), and posterior parietal cortex [1]. M1 generates neural impulses that control the execution of the movement of every body part [1]. Signals from M1 activate contralateral skeletal muscles, which is why stroke patients experience paralysis contralateral to the side of brain damage [1]. In M1, body parts are represented somatotopically (foot, leg, back, arm, hand, etc.) [1]. The amount of brain matter in the motor cortex devoted to any particular body part represents the amount of movement and sensitivity that the body part possesses [1]. This disproportionate “map” is called the motor homunculus [1]. The posterior parietal cortex is involved in transforming visual information into motor commands [1]. For example, the posterior parietal cortex would be involved in determining how to steer one’s arm to a glass of water based on where the glass is located in the space surrounding that individual. The posterior parietal cortex then sends this information to the premotor cortex and the supplementary motor area. The PMA lies anterior to the M1 region. The PMA is involved in the sensory guidance of movement and controls the more proximal muscles and trunk muscles of the body [1]. The SMA lies above, or medial to, the premotor area, also anterior to the primary motor cortex. It is involved in the planning of complex movements and in coordinating two-handed movements. The supplementary motor area and the premotor regions both send information to the primary motor cortex as well as to brainstem motor regions in order for actions to be carried out. In summary, the information goes from the posterior parietal cortex→SMA and PMA→M1.

Motor imagery cannot be mentioned without mentioning mirror neurons. According to a secondary resource in the National Center for Biotechnical Information, mirror neurons are responsible for motor imagery [5]. These neurons fire when the individual imagines, watches, or performs the same action [6]. This confirms the results of a study done by several scientists from The Proceeding of the National Academy of Sciences of the USA. The study measured electrocorticographic cortical surface potentials (electrical activity in the cortex) in eight people during overt action and kinesthetic imagery of the same movements focusing on high and low-frequency ranges and confirmed that the same areas of the cortex were active during overt action and kinesthetic imagery of the same tasks [7]. According to another NCBI study done by Kilner and Lemon, in humans, brain activity consistent with that of mirror neurons has been found in the PMA, the SMA, the primary somatosensory cortex, and the inferior parietal cortex (includes Wernicke’s area) [7]. Of these four regions, the PMA and SMA are located in our motor cortex. There was no mirror neuron activity in M1 [6]. These results support the results of a study by Sharma et al. for stroke and another study done by Dechent et al. from NCBI [1,8]. The stroke study mainly focused on upper limb recovery and functional imaging in healthy patients and those that suffered from a stroke. The study showed that in healthy patients, robust activation of the nonprimary motor structures and weak and inconsistent activation of M1 occur during motor imagery [1,3]. The NCBI study used functional magnetic resonance imaging (fMRI) to reveal that motor execution activated M1 as well as other parts of the motor system including SMA and PMAs [5,8]. In contrast, motor imagery did not lead to activations in M1 except for one of six subjects but involved the SMA, PMA, and the anterior intraparietal cortex [5,8]. 

When analyzing the results of previous studies, it was clear that data was collected using several different instruments and methods. To detect brain activation and connectivity, two consistently used instruments were the MRI and functional near-infrared spectroscopy (fNIRS). MRI, or magnetic resonance imaging, uses a magnetic field and radio waves to produce detailed images of the brain and the brain stem [9,10]. An MRI differs from a computerized axial tomography (CAT) scan because it does not use radiation [9]. fNIR is a brain-imaging technique that uses infrared light to measure changes in oxygenated and deoxygenated hemoglobin levels due to hemodynamic response (the rapid delivery of oxygenated blood to active cortical areas through neuromuscular coupling) [11]. It is portable, unlike fMRI, but has lower spatial resolution and is limited to the cerebral cortex [9,11]. Experiments with fNIRS are designed in the face of a limited number of sources and detectors (optodes) to be positioned on selected portions of the scalp [9,11]. The optodes' locations represent an expectation of assessing cortical regions relevant to the experiment’s hypothesis [10,11]. This can be a challenge, so Morais et al proposed an approach that automatically decided the fNIRS opcodes from a set of predefined positions to maximize the anatomical specificity to brain regions-of-interest [11]. The implemented method is based on photon transport simulations on two head atlases [11]. A prominent statistical method of recording the neurological effect of motor imagery was the Fugl-Meyer Assessment (FMA). The FMA is a stroke-specific, performance-based impairment index [12]. It is designed to assess motor functioning balance, sensation, and joint functioning in patients with post-stroke hemiplegia [12]. We are interested in its ability to describe motor recovery. The FMA scale has five domains: (1) motor functioning in the upper and lower extremities, (2) sensory functioning (evaluated light touch on two surfaces of the arm and leg, and position sense for eight joints), (3) balance (contains seven tests: three seated and four standing), (4) joint range of motion (eight joints), and (5) joint pain [12]. Scoring is based on direct observation of performance: 0 = can’t perform; 1 = performs partially; 2 = performs fully. There are 226 points possible [12]. The motor score (1) in particular ranges from zero to 100 and different papers have different divisions of scores indicating differing levels of motor impairment severity [12]. 

This paper will review previous studies and analyze the brain’s role in motor imagery and the effects of motor imagery on motor execution. Ultimately, the goal of this paper is to determine whether motor imagery is a viable rehabilitation method for individuals who have cortical damage, particularly to the motor cortex.

## Review

Many new studies have analyzed different aspects of motor imagery. They’ve looked into the effects of motor imagery when it was provided as a supplement to traditional physical therapy, the brain’s activation, and connectivity patterns while participating in motor imagery, and the limitations of motor imagery in certain patients.

Motor imagery for motor rehabilitation 

Several studies have looked at the effects of motor imagery in conjunction with established physical therapy methods, such as occupational therapy, and as a physical therapy treatment itself. Fansler et al. assigned 36 elderly women over the age of 70 years to one of three conditions: condition A, control; condition B, relaxation; or condition C, ideokinetic facilitation (an approach to improve posture, balance, and movement fluency using MI) [2]. After comparing the baseline and final measures of one-legged balance time over a three-day intervention period, the investigators had results showing significant improvement between baseline and final conditions within condition C only. The authors concluded that the mental rehearsal of a physical task can enhance performance and may be of use to more clinical approaches [2]. This conclusion is also supported by the results of Linden et al. which reported better equilibrium characteristics in elderly women as measured by walking balance and foot placement measures as a result of a combined treatment of motor imagery and physical therapy [13]. These studies indicate that motor imagery still shows significant beneficial effects in elderly individuals whose neurological abilities are in decline, making them a potential noninvasive method for helping the elderly improve balance and posture during physical activities such as walking. A possible future investigation could look into the effects of aging on motor imagery potential. Another study by Fairweather et al. investigated the effect of MI on muscle pain and postural control. Their study involved a three-week imagery program combined with physical exercise and results showed that the program reduced back pain and improved postural control in patients who suffered from chronic back pain and had varying degrees of lordosis and kyphosis [14]. These studies support the conclusion that beyond motor execution, motor imagery may have beneficial impacts on balance, posture, and possibly chronic pain relief, particularly when used in conjunction with physical therapy. 

Some studies analyzing motor imagery in conjunction with physical therapy were specific to stroke patients. Page wanted to directly test the effect of motor imagery on the requisition of function following stroke [15]. In his study, eight patients who had right-arm hemiparesis were put through a four-week program that combined occupational therapy and MI while eight controls were put through a program only consisting of occupational therapy. Occupational therapy (OT) is a type of physical therapy focused on developing skills specific to an individual’s line of work [15]. Using the Fugl-Meyer Assessment of Sensorimotor Recovery, they found that those who went through the OT+MI program exhibited significantly more improved function than those who just received OT [15]. This study shows that MI can be a non-invasive and inexpensive compliment to OT, enhancing the resulting motor improvement in its subject [9]. Liu et al. conducted a similar study, which involved 26 stroke patients that received MI in combination with physical therapy for three weeks(one hour per day). Results showed that patients who received motor imagery as part of their treatment improved significantly more on functional tasks than the patients who only received additional assistance from the therapist [16]. Although both studies present promising results, they were both aimed at learning the movements involved in their daily activities, and not relearning basic motor skills [9]. This may be why the patients improved on the specific tasks of their daily activities or work but not necessarily on motor performance as a whole, which relies on improving the foundation: basic motor skills [9]. Nevertheless, Liu et al. also reported that the patients in the motor imagery group also improved on neuropsychological tasks measuring attention, suggesting that motor imagery training may have a beneficial impact on one’s attentiveness as well [16]. 

It should be noted that all the patients in the study by Page were in the early post-stroke period (two to 11 months). Thus, another study by Page et al. in 2005 looked into whether or not motor imagery is useful in chronic stroke patients. Six patients with a post-stroke period greater than one year were trained for six weeks with a combination of physical therapy and motor imagery, while a control group received physical therapy and relaxation exercises. The results indicated that arm function improved more for the patient group than the control group [17]. Although more studies need to be conducted analyzing motor imagery in chronic stroke patients, this particular study supports the conclusion that motor imagery may play a therapeutic role even in patients more than a year past stroke. Additionally, this study and Fansler et al. both show that relaxation is consistently having little to no impact compared to MI on motor execution improvement which adds to the recurring theme that mental stimulation - with or without motor execution - is more effective than mental relaxation. Cincotta et al. supports this conclusion [2,17,18]. Their study investigated the effect of MI on motor evoked potential (MEP) in quadriplegic patients diagnosed with locked-in syndrome, a rare neurological disorder in which there is complete paralysis of all voluntary muscles except for ones that control the movement of the eye [18]. Stroke electromyography (EMG) and transcranial magnetic stimulation(TMS) recordings revealed no motor activity in the right abductor digiti minimi (ADM) or either tibialis anterior muscles [18]. There was some MEP from the left ADM, however, they had prolonged latency and reduced amplitude [9]. After the patients were instructed to imagine the movement of their paralyzed fingers, the latency, and elicitability significantly improved compared to the control. The results of this study are significant because they show that MI can be used to gain some control over a peripheral effector without being able to compare MI to ME [9,18]. By analyzing the strength of MEP before and after MI, the results show that MI has an impact even in those with the most severe cases of paralysis and as a result could lead to new rehabilitation methods for these patients. The results also showed that even when there is stroke damage present and the movement of an area is lost, the neuromuscular pathway is not completely severed, a finding supported by Pool et al. and Wang et al. whose studies indicated that although lesioned cortical areas will cause abnormal network activity during motor execution in patients, they have no significant effect on motor imagination [19,20]. This conclusion is also indirectly supported by Raffin et al. whose study aimed to investigate whether amputees can distinguish between executing a movement of the phantom limb and imagining the movement of the missing limb [21]. They examined the capacity of 19 upper-limb amputees to execute and imagine movements of both their phantom and intact limbs and compared the results to that of 18 age-matched normal controls. Amputation reduces the speed of voluntary movements with the phantom limb but does not change the speed of imagined movements, suggesting that the absence of the limb specifically affects the ability to voluntarily move the phantom limb but does not change the ability to imagine moving the missing limb [21]. In other words, the potential of MI is mostly independent of the loss of motor execution. This is probably because - as reported in Dechent et al. and Hetu et al. - MI consistently recruits a large frontoparietal network, subcortical and cerebellar regions, the anterior intraparietal cortex, and the bilateral SMA and PMA [8,22]. Furthermore, Hetu et al. reported that motor imagery leads to activation in the M1 region for only one-sixth of their subjects, indicating that the activation may have been due to confounding variables and not actually MI [22]. Since motor execution is significantly lost from stroke damage to the M1 region, motor imagery largely remains near its potential before brain damage. A future study should directly compare MI potential in patients with a range of motor cortical damage, primarily with the M1 region. 

Brain activation during motor imagery

Several studies have identified patterns in brain activation that occur during motor imagery. Dechent et al. and Hetu et al. found that MI largely activates regions including the frontoparietal network, subcortical and cerebellar regions, the anterior intraparietal cortex, and the bilateral SMA and PMA [8,22]. Analyzing these patterns and comparing them to those present during motor execution is key to discovering the potential of motor imagery, especially in individuals with cortical damage. Wang et. al. recorded which cortical regions were active during motor execution and motor imagery in a set of 10 patients with right-hand hemiplegia and 10 controls who did not have any form of paralysis [20]. Using magnetic resonance imaging, they were able to present the location and intensity of active cortical regions in the subjects during motor execution (ME) and motor imagery (MI). The results showed that, in the controls, cortical motor areas were activated during both motor execution and motor imagery, particularly the PMC, M1, and SMA [20]. However, the intensity of the activation during imagery was less than that during execution [20]. Additionally, the overall cortical activation in patients was lower in intensity than the activation in the controls [20]. This is expected because of the brain lesions in patients due to stroke damage [20]. The important point to note is that much of the same motor cortical regions were activated during motor imagery and motor execution. 

Correlations in the activation patterns were also noted by Mizuguchi and Kanosue, whose investigation looked into how neural mechanisms differ across the performance spectrum of athletes, from beginner to expert. They observed that during both action observation and motor imagery, the same set of motor-related regions such as the premotor cortex and inferior parietal lobe were activated [23]. Their results also showed that activation in motor-related areas during action observation and motor imagery was higher in experts than in nonexperts, while activation during motor execution was often lower in experts than in nonexperts [23]. This is because brain activation is influenced by task complexity [4,9,24]. The experts analyze and think about the technique and how to execute the particular movement more intricately than non-experts, thus their activation is higher during motor imagery and action observation [4]. When executing the movement, experts act with more ease due to more training and muscle memory, thus their activation during ME is lower than that of nonexperts. These results suggest that being trained in physically complex movements - be it through sports, one’s line of work, or even playing an instrument - may contribute to improving the potential of one’s motor imagination. Future studies should investigate and compare how different physical activities influence the MI capabilities of experts in each of those activities. 

**Figure 1:**
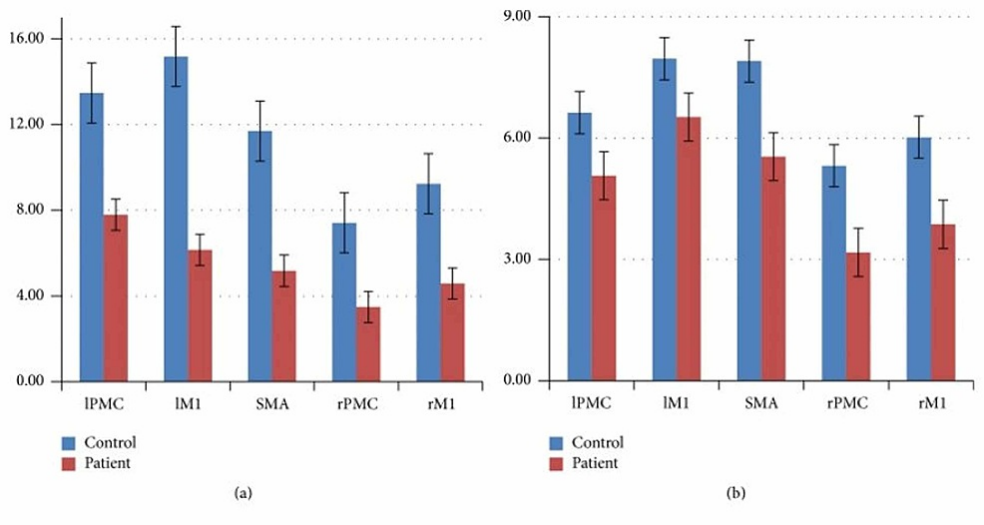
The statistical analysis of the mean value of the highest positive t value of the subject-specific ROI in the two groups, (a) motor execution; (b) motor imagery. Source: Wang et al. [20] IPMC: left premotor cortex; lM1: left primary motor cortex; SMA: supplementary motor area; rPMC: right premotor cortex; rM1: right primary motor cortex; ROI: region of interest

Although there may be overlap and similarities in what regions ME and MI activate, there are more distinguishable differences as each cortical hemisphere and region is examined. Wang et al. found that contralateral motor regions were more strongly activated than their ipsilateral motor regions in controls as expected [20]. As mentioned earlier, in the patient group, overall motor cortical activity had a lower intensity than the controls during both motor execution and motor imagery [20]. However, unlike the controls, there was not a significant reduction in cortical activity from ME to MI. Thus, as shown in Figure 1, the difference between the control group and the patient group in the intensity of activation of their different motor cortical areas is much smaller during motor imagery than motor execution. 

Wang et al. found that in addition to the motor cortical regions, stroke patients also showed significant activation in the frontal and parietal lobe [20]. This is supported by Hetu et al., whose investigation combined the data from 75 papers and determined that MI consistently recruits a large frontoparietal network in addition to subcortical and cerebellar regions [22]. The intensity of the activation of motor cortical areas for the patients was very close - mostly within the margin of error - when comparing motor imagery and motor execution [20]. For the controls, on the other hand, the intensity of activation during motor imagery was significantly less than their intensities during motor execution. As a result, there was a much smaller difference in activation between the controls and patients during motor imagery compared to motor execution. These results suggest that the cortical damage had a much smaller impact on MI than ME, thus showing that motor imagery is a viable method of activating cortical regions regardless of whether there are existing brain lesions.

Additionally, Figure 2 from Wang et al. makes it visually clear that the area of brain activation in the right hemisphere during motor imagery in the patients was actually larger than during motor execution. This emphasis on ipsilateral activation showcases the brain’s ability to functionally reorganize its neural networks. Furthermore, the finding that the frontal and parietal lobes were significantly activated in the patients shows the reorganizational capability of the brain. Since there were parts of the motor cortex that could not be activated due to damage, the brain recruited other regions involved in the planning and control of limb movements. This is a prime example of the potential of brain plasticity and activity reorganization

**Figure 2:**
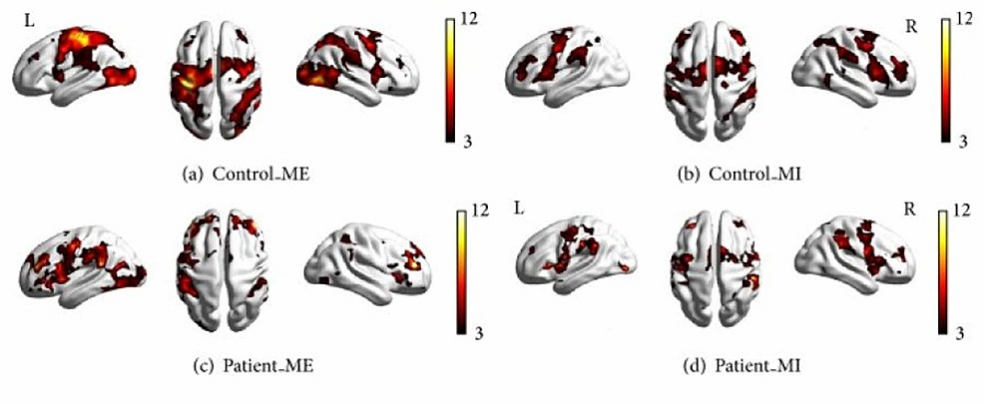
Brain activation in the control and patient groups under different conditions. (a) Control subjects during motor execution; (b) controls during motor imagery; (c) patients during motor execution; (d) patients during motor imagery. All voxels were significant at p < 0.01, corrected for FDR at the whole-brain level according to Wang et al. [20]. ME: motor execution; MI: motor imagery; FDR: false discovery rate

**Figure 3:**
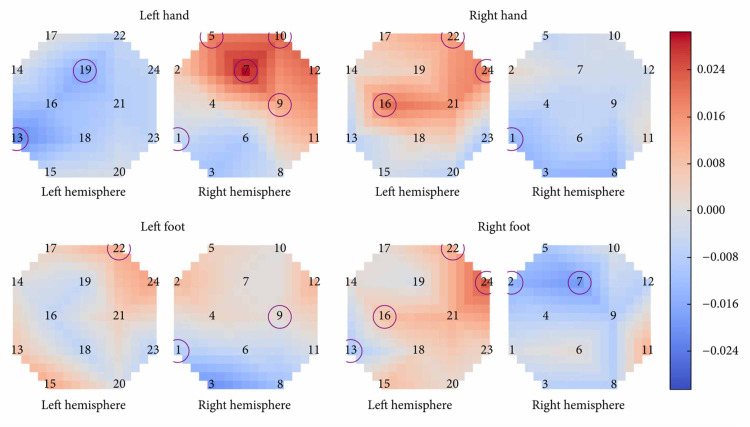
Average difference in activation between motor execution and motor imagery. Optodes with a significant difference (p < 0.05, FDR adjusted) between motor execution and motor imagery for a given task are circled according to Batula et al. [10]. FDR: false discovery rate

Batula et al. compared the cortical activation patterns of MI and ME in upper and lower limbs using fNIRS, a brain-imaging technique that uses infrared light to measure changes in oxygenated and deoxygenated hemoglobin levels due to hemodynamic response(the rapid delivery of oxygenated blood to active cortical areas through neuromuscular coupling) [9,10]. Unlike fMRI, fNIRS is portable, but has lower spatial resolution and is limited to the cerebral cortex [9]. Thirteen healthy, right-handed participants were instructed to perform five tasks: rest and four simple finger- and toe-tapping actions(right arm, left arm, right leg, and left leg). The FNIR detected significant activation during motor imagery of all four motor tasks [10]. Results showed that compared to MI, ME produced a faster response, a different spatial distribution of activation, and higher activation levels [9,10]. The timing of hemoglobin O (HbO) levels increasing was slower during MI activation than during ME activation [10]. S.C. Wriessnegger et al. reported a similar finding when their subjects showed significant increases in oxy-Hb during both MI and ME of right and left hand tapping tasks compared to the resting period, but with different onset latencies of oxygenation [25]. Results specifically showed that during left and right MI, the oxy-Hb concentration increased about two seconds slower than ME [25]. Although further investigation is needed, this finding highlights a unique difference between ME and MI revealing the relationship between the speed of hemodynamic response and the complexity of the motor activity the brain is pursuing. Batula et al. also found that ME and MI had different spatial distributions [10]. Figure 3 has circles representing optode locations and the squares on each graph represent a particular cortical area [10]. The color of each square depends on the value after subtracting MI from ME [10]. Knowing this, we can notice how MI had higher ipsilateral/bilateral activation while ME had higher contralateral activation. The left hand and right foot had an especially high activation pattern during motor execution, and this may in part be because the subjects were all right-handed, so using their left hand was unnatural and thus required more brainpower, literally [10]. Upper limb tasks were more easily distinguishable(spatiotemporal activation patterns) while left and right lower limb activation patterns were found to be highly similar during both imagery and execution, leading the investigators to believe that higher resolution imaging, advanced signal processing, or improved subject training may be required to reliably distinguish them [10]. The right hand demonstrated mostly contralateral activation patterns for both motor imagery and motor execution conditions, as shown in Figure 3, while the left hand showed a much more bilateral activation during motor imagery [10]. The left hand also had more contralateral ME activation. This could be due to the fact that all participants were right-handed, potentially making the right-hand task easier to imagine and execute. Lower limb tasks had more bilateral HbO activation patterns than upper limb tasks in both ME & MI [10]. This may be because toe-tapping takes more mental effort and precision of fine motor skills than finger-tapping since toe-tapping is not a commonly executed movement. There was also less of a distinction in the activation patterns of the lower limbs [10]. This may be because toe and foot motor areas are near or within the longitudinal fissure between brain hemispheres, which is more difficult to measure, while leg motor areas are further apart and closer to the surface of the scalp [10]. One solution to this may be to use full leg movement instead of just toe-tapping, as done by Hsu et al. [26]. Finally, just like the results from Wang et al., ME showed higher activation than MI overall. Significant differences between ME and MI have also been reported in Sitaram et al. and Wreissnegger et al. [25,27]. These significant differences may partially be due to the continuous somatosensory and visual feedback of the movement and muscle stimulation that is only present during motor execution [1,27,28,29].

Connectivity

**Figure 4:**
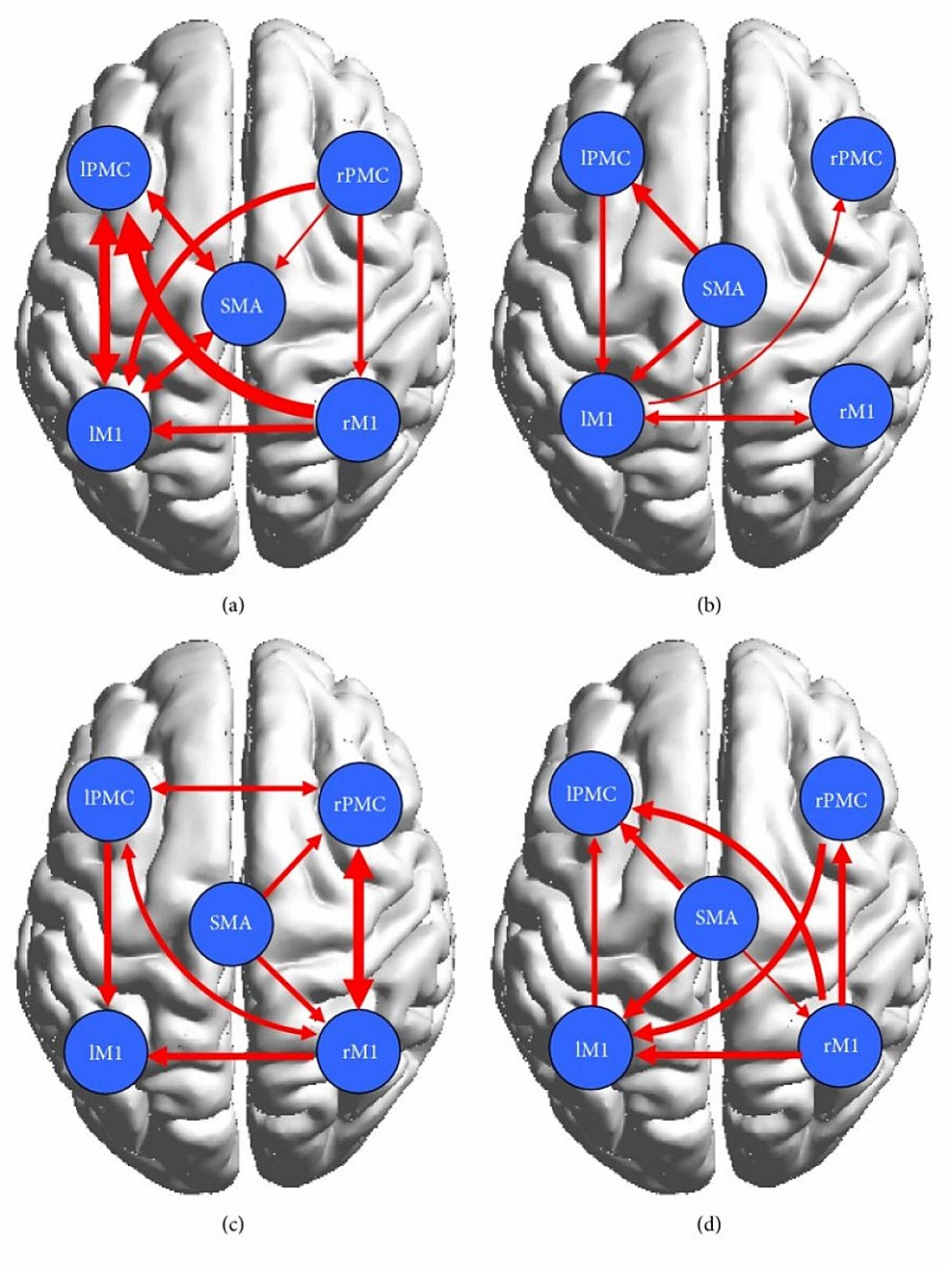
(a) and (b) display significant effective connectivity within the cortical motor network in the healthy controls during ME (a) and MI (b). (c) and (d) display significant effective connectivity within the cortical motor network in the patients during ME (c) and MI (d). The thickness of the lines is proportional to the connection strength according to Wang et al. [20]. ME: motor-execution; MI: motor imagery; IPMC: left premotor cortex; lM1: left primary motor cortex; SMA: supplementary motor area; rPMC: right premotor cortex; rM1: right primary motor cortex

Wang et al. found that in the control group represented in Figures 4a, 4b, the cortical network showed connectivity among the contralateral regions during both motor execution and motor imagery, however, they were significantly lower during motor imagery [20]. Researchers found that effective bilateral connectivity was observed in contralateral regions during motor execution but not motor imagery [20]. This is understandable since motor imagery does not require too much activation of areas of the brain involved in analyzing one’s surroundings while they are performing the movement [7]. In the patient group, which is represented by Figures 4c, 4d, stronger ipsilateral connectivity was observed. Using the conditional Granger causality analysis (CGCA), a statistical hypothesis test, researchers found that there was a decrease in effective connections in the damaged cortical area, but they also found that there were more effective connections between the non-damaged, mostly ipsilateral, brains due to the information loop between the SMA and ipsilateral PMC and M1 as seen on Figure 4c below [20]. Thus a more complex effective connective motor network was observed in the patients during motor imagery, as displayed by the connections shown in Figure 4d. The decrease in connectivity and any abnormal brain connections during motor execution can be attributed to brain lesions, as referenced by other studies [1,4,25,26,29]. Furthermore, as shown by Figure 4a, although a bidirectional connection between the left primary motor cortex (LPMC) and LM1 was present in the control group, only a unidirectional connection from LPMC to LM1 was shown in the patient group represented by Figure 4c [20]. It was concluded that a lesion was responsible for the absent connection from LM1 to LPMC. All in all, the overall ipsilateral connectivity in the patient group is actually stronger, although their effects may not be of the same magnitude, as the control group. Other studies have reported, in fact, that stroke patients activate more ipsilateral cortical areas to perform simple motor tasks [20]. The data from the Wang et.al paper supports that conclusion as can be seen by the bilateral connection between the right PMC and M1 and the unilateral connection between the SMA and right PMC and M1, all of which have weaker connections in the control group. The strengthening of ipsilateral connectivity among non-damaged cortical areas is a testament to the brain’s plasticity and its ability to reorganize the areas it is recruiting to fulfill motor tasks.

**Figure 5:**
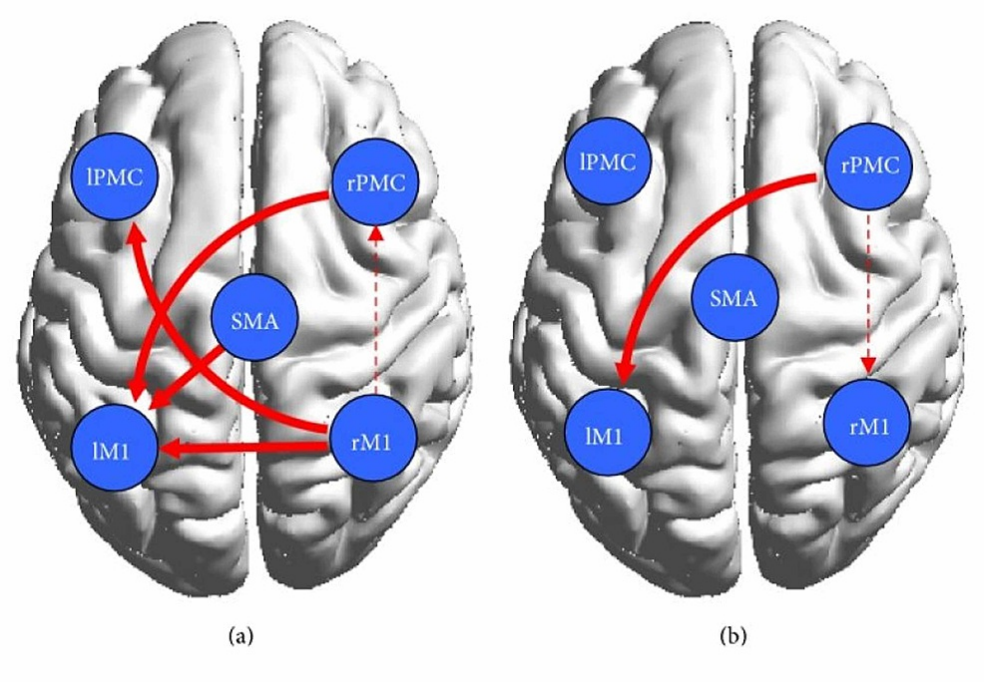
Results of the motor network causal interactions in the controls versus the stroke patients during ME and MI. Solid lines indicate significantly stronger connections in the control group than in the patient group, and dashed lines indicate the opposite according to Wang et al. [20]. ME: motor-execution; MI: motor imagery; IPMC: left premotor cortex; lM1: left primary motor cortex; SMA: supplementary motor area; rPMC: right premotor cortex; rM1: right primary motor cortex

To further study and compare the causal relationship within the motor cortex between the control and patient groups, the researchers implemented a two-sample t-test [20]. In Figure 5, which shows the result of the test, the solid red lines represent significantly stronger connections during ME and the dotted red lines represent significantly stronger connections during MI [20]. Evidently, the test revealed that the bilateral interaction during motor execution was much weaker in the patients, which was expected due to the presence of cortical damage. The control group had stronger interactions between the left PMC and the right M1 and the right PMC and left M1. During motor imagery, there were tighter cortical motor connections in the patients than in the controls. In fact, the effective motor connectivity in the patients was actually enhanced during motor imagery. Additionally, Pool et al. found that stronger coupling between the contralateral SMA/PMA and the M1 could enable increased motor performance in unilateral hand movements in healthy people [19]. These interactions were not observed during motor execution in patients, however, they were observed during motor imagery, thus indicating that although lesioned cortical areas will cause abnormal network activity during motor execution in patients, it has no significant effect on motor imagination, a pattern that was observed in with brain activation in the Wang et. al study as well. The increased effective interactions during motor imagery support the conjecture that motor imagery enhanced bilateral connectivity in the stroke patients and, as a result, may have facilitated partial motor function recovery.

**Figure 6:**
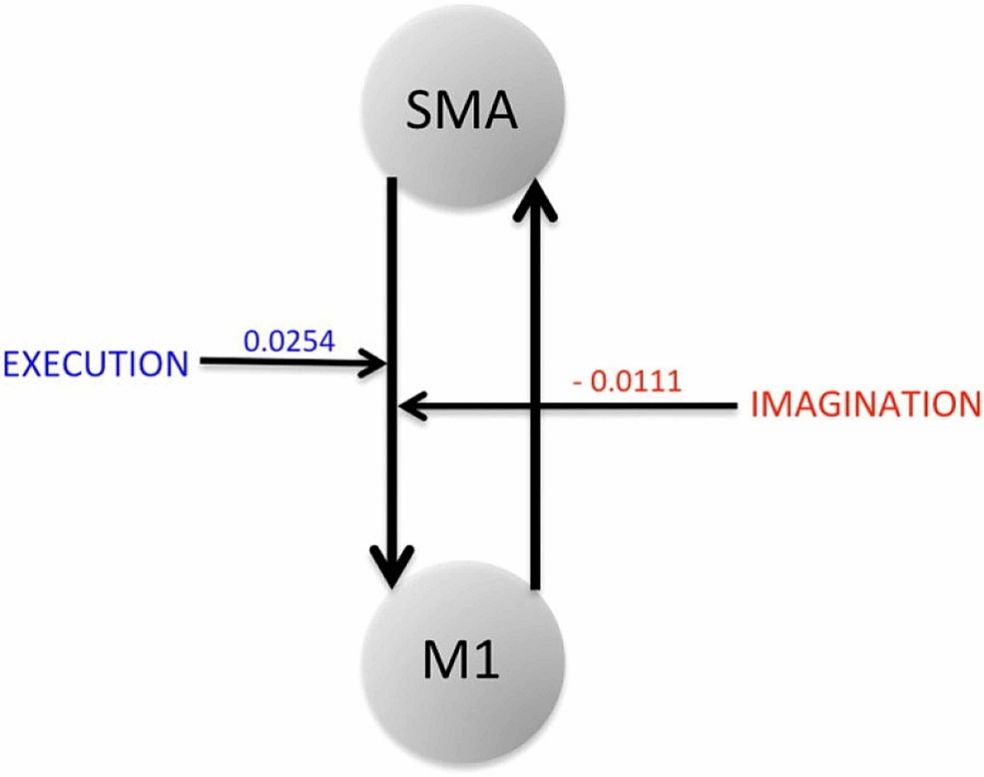
SMA to M1 connection is positively modulated during ME whereas the same connection is negatively modulated during MI. Source: Bajaj et al. [30] SMA: supplementary motor area; M1: primary motor cortex; ME: motor-execution; MI: motor imagery

Bajaj et al. looked at how brain network interactions reorganize and recover their functionality during recovery and treatment following a stroke, by comparing the connectivity between affected and unaffected hemispheres [30]. They recorded blood oxygenation-level dependent (BOLD) fMRI signals from 10 stroke survivors and evaluated dynamical causal modeling (DCM)-based effective connectivity among three motor areas: M1, PMC, and supplementary motor area (SMA) during ME and MI tasks [30]. After giving the patients 60 hours of intervention, they made a few key findings. Using the Bayesian model averaging (BMA) approach, they found that the intervention improved the regional connectivity among the motor areas during both ME and MI [30]. The connectivity between PMC and M1 was stronger in MI tasks whereas the connectivity from PMC to M1, SMA to M1 was stronger in ME tasks [30]. Furthermore, the Fugl-Meyer Assessment (FMA) showed that there was a significant behavioral improvement (p = 0.001) in sensation and motor movements because of the intervention [12,28]. Finally, they found that the same network dominated during motor-imagery and motor-execution tasks but modulatory parameters suggested a suppressive influence of SMA on M1 during the motor-imagery task whereas the influence of SMA on M1 was unrestricted during the motor-execution task, as shown by Figure 6 [30]. Due to this interaction with the SMA, M1 causes more exchange of causal information among motor areas during a ME task than during an MI task [30]. These findings suggest that the interaction between the PMC and M1 plays a crucial role during ME and MI.

The limitations of motor imagery

Motor imagery has shown promising results in retaining lost motor capabilities in patients with cortical damage. However, we must also consider the possibility of cases where patients are not able to imagine motor movement due to a large extent of brain damage. At what point are enough mirror neurons damaged that motor imagery would not be viable to even pursue? What factors compromise the capabilities of one’s motor imagination?

Some of the earliest papers on motor imagery began emerging in the 1990s and a primary focus of those studies was to understand what motor imagery even was and what neural pathways contributed to its functioning. In order to do this, those researchers investigated where motor imagery took place in the cortex and what its limitations were. Jackson et al. found that lesions in the parietal lobe may impair motor imagery [31]. This correlates with the findings in Dechent et al. and Hetu et al. that MI largely activates regions including the frontoparietal network, subcortical and cerebellar regions, the anterior intraparietal cortex, and the bilateral SMA and PMA [8,22]. The study by Lotze & Halsband also supports this finding as their results showed that patients with parietal lesions and left lateral prefrontal lesions are not able to imagine movement [32]. Sirigu et al. found that patients with lesions in the parietal cortex were found to be impaired selectively at predicting, through mental imagery, the time necessary to perform differentiated finger movements and visually guided pointing gestures in comparison to normal individuals and to a patient with damage to the primary motor area [33]. These results suggest that the parietal cortex is important for the ability to generate mental movement representations. 

Other studies investigated the potential of MI in patients diagnosed with neurodegenerative diseases, such as the study done by Yaguez et al. which involved patients with Parkinson's and Huntington's [34]. Huntington's disease (HD) causes degeneration of parts of the basal ganglia, specifically the caudate nucleus and putamen [34]. Parkinson's disease(PD) degrades the basal ganglia and substantia nigra, whose neurons are responsible for producing the neurotransmitter dopamine and for relaying messages that plan and control body movement [34]. These two diseases were probably of interest because they both affect the basal ganglia, which is responsible for motor control as well as roles in motor learning. Yaguez et al. compared the ability of two groups, 11 non-demented mildly affected HD patients and 12 non-demented PD patients, to learn a graphomotor task. The researchers took a baseline measurement, provided patients with 10 minutes of MI, and then had them motor practice [34]. Results showed that MI alone improved the motor performance of the graphomotor task in HD patients but MI nor motor practice had any effect on the PD patients’ ability to perform the graphomotor movement [34]. The PD patients also had more difficulty solving the visual imagery tasks while the HD patients’ performance with the visual imagery tasks was shown to have a correlation with the degree of atrophy in the caudate nucleus [34]. Since there appears to be no correlation between the performance of visual imagery and the improvement of motor performance through MI, the results indicate that motor imagery and visual imagery and independent processes, which are supported by Jackson et al. [9,28,31]. By showing the significant effects Parkinson's has MI, the results suggest that the dopaminergic input to the basal ganglia plays an important role in the translation of motor representations into motor performance [9]. The results also suggest that while the degree of atrophy in the caudate nucleus of HD patients does not affect their ability to perform MI, it does affect their visual imagery (VI) process [34]. This may be because of the caudate nucleus’s unique role in vision coordination. According to Hikosaka et al., the caudate nucleus integrates visual input and inhibits the substantia nigra, disinhibiting the superior colliculus to enable the coordination of eye movement, and is important in voluntary saccadic eye movement [35]. Since visual imagery completely relies on the subject’s perception of the movement, the degree of atrophy to the caudate nucleus would presumably have a negative effect on their VI capabilities, which is what happened in this study [28,34,35]. Furthermore, the deficits found in PD patients might also be due to their limited attentiveness and difficulties in employing predictive motor strategies [9]. This was seen in the study by Berardelli et al. whose results indicated an association between Bradykinesia - a symptom of PD that causes slowness in movement - and one’s ability to perform MI [36].

Looking further into the effect of PD on MI, Thobois et al. conducted a PET study that investigated brain activation abnormalities of PD patients during MI and whether MI activation patterns depend on the hand used to complete the task [37]. The study was performed on eight PD patients with predominantly right-sided akinesia and eight age-matched control subjects who were all right-handed. Regional cerebral blood flow was measured while the patients engaged in specific MI tasks. In normal subjects, the prefrontal cortex, supplementary motor area(SMA), superior parietal lobe, inferior frontal gyrus, and cerebellum were activated during motor imagery with either the left or the right hand [37]. Contralateral primary motor activation was noted only when the task was imagined with the right(dominant) hand, whereas activation of the dorsolateral prefrontal cortex was observed only during imagery with the left hand [37]. When motor imagery with the right akinetic hand was tested in the PD patients, there was a lack of activation of the contralateral primary sensorimotor cortex and the cerebellum, persistent activation of the SMA, and bilateral activation of the superior parietal cortex [37]. Motor imagery with the left non-akinetic hand was also abnormal, with a lack of activation of the SMA compared with controls [37]. In summary, in PD patients with right-sided akinesia, brain activation during MI is abnormal and appears with the less affected hand as well [37]. In normal subjects, brain activation during MI is affected by the hand being used [37]. Thobois et al. support the previous studies in showing that PD does play a negative role in MI capability. 

Lim et al. studied the effect of visual and motor imagery on the contingent negative variation (CNV) in PD patients and in a group of age-matched controls [38]. CNV is a steady, slow, negative-going sensory-related waveform often observed in the central and frontal areas and is considered a response-locked measurement [16], which means that the epochs are cut around the time the participant took to respond to the task instead of cutting it relative to the onset of the stimulus [38]. CNV has two main components: early and late [9,38]. The early component is frontally distributed, specifically involving the prefrontal cortex and cingulate motor areas [38]. The late component is generally by the basal-ganglia thalamocortical loop and reflects activity in the primary motor cortex [38]. Lim et al. investigated whether MI would alter the movement-related potentials with PD [38]. They expected motor imagery to have a larger effect on the CNV than visual imagery [38]. Results showed that 10 minutes of visual imagery had no effect on the amplitude of the CNV in PD patients are the controls [38]. The MI increased the amplitude of CNV in PD patients but had no effect on the controls [38]. The authors concluded that MI could be a promising method for motor rehabilitation in PD patients, which contradicts the findings of Yaguez and Thobois [34,37,38]. One reason for the difference in results may be that Lim et al. measured the CNV of specific cortical regions while the other two studies looked for activation in more general areas, such as the cerebellum. Additionally, different methods of measurement were used, and so the PET scan and CNV may have different sensitivities and thus indicate different results. Nevertheless, more studies need to compare the MI ability in PD patients with normal controls as data right now is very limited. 

We’ve seen how patients with cortical damage can use MI to activate partially damaged motor networks and facilitate functional reorganization within their brains. Studies have shown that this process is compromised in cases with right posterior or left frontal lesions. Johnson stated that this finding is consistent with the hypothesis that imagined prehension - a reaching and grasping movement - involves a network of highly interconnected areas distributed throughout the parietal and frontal cortices [39]. Earlier studies involving hemiplegic subjects suggest that they may retain the ability to generate images of movements they no longer can perform [9]. However, these results were based on introspection and questionnaires which are subjective methods and can thus vary from subject to subject [9]. Johnson used a more objective assessment to investigate the MI capability of hemiplegic patients [39]. The task was for the patient to think of the most efficient handgrip to execute a particular action [39]. By solving this problem, they were effectively using MI [9,39]. Results showed that there were no differences between hemiplegic patients and healthy ones in solving this task [39]. These results are significant because they show that hemiplegic patients are still able to have MI capabilities on par with controls with no brain lesions, further supporting a theme among many papers that MI and ME capabilities are independent processes. Future studies should assess activation patterns in addition to imagery tasks so we can investigate whether a difference in activation in hemiplegic patients during MI and to what degree this difference actually affects their imagination ability as compared to the control group.

The majority of individuals who experience a stroke are over the age of 65 years [9]. Therefore, as was mentioned in an earlier section of this paper, studying the effect of age on MI capability is relevant when assessing the viability of MI. Mulder et al. conducted a study using a questionnaire that scored three hundred 33 participants on their vividness of movement imagination [40]. Participants were divided into three age groups: 30 years, 30-60 years, and greater than 64 years [40]. Results showed that elderly participants were slightly worse in their motor imagination capabilities than younger participants, particularly in relation to MI from an internal(first person) perspective [40]. However, since the difference was slight, it may just be a natural symptom of age. Further studies need to compare MI capabilities of participants who all have stroke damage. This would give us a clearer picture as to whether the differences in MI capabilities in each of the age groups are affected by stroke damage. As of now, there is not enough research done on the relationship between age and MI to make any substantial conclusions. All in all, more investigation is needed on all fronts in order to determine what degree of brain damage compromises MI and what factors outside cortical damage, such as age, physical activeness, and genetic mutations(i.e. Huntingtons) may contribute to differences in MI potential. 

## Conclusions

This review aimed to analyze whether motor imagery may be a viable motor rehabilitation treatment for individuals with motor cortical infarction. Studies investigating the role of MI in rehabilitation treatment have shown that MI has significant beneficial effects on elderly individuals’ balance, posture, chronic pain relief, and other physical abilities. MI has also shown to be a beneficial supplement to occupational therapy and even improve motor evoked potentials (MEP) in patients with the most severe cases of paralysis and chronic stroke damage. These findings ultimately reveal that MI is largely independent of ME and when the movement of an area is lost, the neuromuscular pathway is not completely severed. Additionally, the studies showed that mental relaxation has little to no impact, compared to MI, on ME improvement, thus adding to the recurring theme that mental stimulation - with or without motor execution - is more effective than mental relaxation. This was further confirmed when analyzing the activation and connectivity patterns present during MI and comparing them to those present during ME. As expected, the cortex showed higher activation during ME than during MI, however, there was a much smaller difference in activation between controls and patients during MI compared to ME. These results suggest that the cortical damage had a much smaller impact on MI than ME, thus showing that motor imagery is a viable method of activating cortical regions regardless of whether there are existing brain lesions. Results from multiple studies showed that the ipsilateral and bilateral neural pathways were stronger in patients with stroke damage compared to the controls, thus emphasizing the human brain’s plasticity and ability to functionally reorganize its neural connections. Current studies suggest that cortical damage to the parietal lobe leads to weaker MI capabilities. Additionally, patients with Parkinson’s have shown to have a significantly weaker motor imagination, suggesting a significant role of the basal ganglia in MI. 

More investigation is needed on all fronts in order to determine what degree of brain damage compromises MI and what factors outside cortical damage - such as age, physical activeness, and genetic mutations (i.e. Huntingtons) - may contribute to differences in MI potential. Nevertheless, the findings analyzed in this review indicate that MI would serve as a beneficial addition to physical therapy and be a viable option to stimulate MEPs in individuals not capable of pursuing physical therapy. Regardless of the presence of brain lesions, MI has only ever had a positive impact on motor rehabilitation either in boosting treatment or stimulating neuromuscular pathways. Therefore, we can conclude that motor imagery is a viable supplemental treatment plan for motor recovery in most patients with motor cortical atrophy.
